# A Single-Center Assessment of the Race Neutral eGFR Calculation and Access to Kidney Transplantation for Black Patients: A Policy Change Is Not Enough

**DOI:** 10.1007/s40615-025-02573-9

**Published:** 2026-03-11

**Authors:** Joy E. Obayemi, Ateh E. Fonteh, Daniela P. Ladner, Amishi Desai, Dinee C. Simpson

**Affiliations:** 1 Comprehensive Transplant Center (CTC), Northwestern University Transplant Outcomes Research Collaborative (NUTORC), Northwestern University Feinberg School of Medicine, Chicago, IL USA; 2 Department of Surgery, University of Michigan, Ann Arbor, MI USA; 3 Northwestern University Feinberg School of Medicine, Chicago, IL USA; 4 Division of Transplantation, Department of Surgery, Northwestern Medicine, Chicago, IL USA; 5 Division of Nephrology, Department of Medicine, Northwestern Medicine, Chicago, IL USA; 6 Division of Transplantation, Department of Surgery, Feinberg School of Medicine, Northwestern University, 676 N St. Clair, Suite 1900, Chicago, IL 60611 USA

**Keywords:** Estimated glomerular filtration rate (eGFR), Kidney, Transplant, Race, Disparities

## Abstract

**Introduction:**

Effective July 2022, kidney transplant centers were required to adjust waiting time for Black candidates using a new race-neutral eGFR (estimated glomerular filtration rate) calculation. Little has been reported about the impact and limitations of this policy change on transplant access for Black patients.

**Methods:**

A retrospective, single-center, cross-sectional study was performed in 04/2024 on adult (> 18 years) kidney transplant candidates who were eligible for wait-time modification. Clinicodemographic data and adjusted dialysis start dates were extracted from the medical record. Descriptive statistics were performed.

**Results:**

A total of 274 Black patients were eligible for a wait-time modification. Of these, 26 (9.5%) had been delisted, nine (3.3%) were deceased, 26 (9.5%) were already transplanted, and 104 (38.1%) did not have the required laboratory documentation available. In total, 109 (39.8%) candidates ultimately received wait-time modifications. Of the 109, mean age was 56 years old and 47 (42.7%) were female. Mean wait-time gained was 2.7 years (median, 1.7 years; range, 1 month–21 years). Total wait-time gained by Black patients at this center was 298.5 years. Patients who did not receive time back were more likely to have recent bloodwork from maintenance dialysis as the source of evaluated laboratory data (52.7% vs. 33.0%) (*p* < 0.001).

**Conclusion:**

Removing race from the eGFR calculation expanded transplant access for Black patients with eligible patients receiving an average of 2.7 years of additional wait time at a large metropolitan center. However, wait-time recovery was limited by the availability of laboratory data, indicating that assistance with finding older lab values may help realize the full potential of this policy.

## Introduction

### Historical Perspective

Glomerular filtration rate is the gold standard for assessing a patient’s burden of kidney disease [1]. Given that it cannot be easily measured, estimated glomerular filtration rate (eGFR) is used in its place to synthesize data and inform healthcare providers’ treatment of renal pathology [1]. In 1999, the Modification of Diet in Renal Disease (MDRD) Study published the first widely used equation to calculate eGFR that incorporated six variables: serum creatine level, age, sex, race (African American vs. non-African American), blood urea nitrogen, and albumin [2, 3]. The inclusion of race was justified with the notion that “on average, black persons have greater muscle mass than white persons” [3]. This notion—or rather non-evidence-based misconception—laid the foundation for the systematic over-estimation of renal function for Black patients that persisted for decades.

In 2000, the equation was simplified to include only four variables (creatinine, age, gender, race) and in 2006, the weights of those variables were refined [4]. The MDRD equation was succeeded by the CKD-EPI (Chronic Kidney Disease Epidemiology Collaboration) equation in 2009 and by the combined creatinine and cystatin-C-based CKD-EPI equation in 2012, both of which improved upon the accuracy in certain subgroups [4, 5]. However, throughout the evolution of this equation, the inclusion of race—specifically Black or African American race—remained unchanged. During this time, the MDRD equation assigned a 21% increase in eGFR to all Black patients, the 2012 CKD-EPI equation assigned an increase of 16%, and the 2012 combined creatinine and cystatin-C CKD-EPI equation assigned an increase of 8% [5].

As scholars across medicine and in fields such as sociology, anthropology, and epidemiology began to critically examine the use of race in medical practice, the validity of this racial correction was increasingly scrutinized [6, 7]. Given that race is a socially constructed and geopolitically informed concept—and given the disproportionately high rates of renal failure among Black Americans[8]—concern was raised that this racial correction was contributing to the inequitable access to specialty care and kidney transplantation observed among Black patients [9, 10]. It is important to note that significant variations in referral patterns exist and barriers to prompt referral are abundant, a full discussion of which is beyond the scope of this text [11]. However, generally speaking, a referral for kidney transplantation may be placed when the patient starts dialysis or when a patient’s eGFR drops below 20 mL/min per 1.73 m^2^. The inclusion of race in the eGFR equation resulted in a falsely elevated eGFR, which further contributed to a systematic delay of referral for kidney transplantation and waitlisting of Black patients [12].

### Current Situation

In 2020, amidst mounting pressure to eradicate race-based calculators and other vestiges of structural racism embedded in the healthcare system, leaders from the National Kidney Foundation and the American Society of Nephrology created a joint task force consisting of 97 experts and patients to discuss the evidence and role of race in the eGFR calculation [13, 14]. In what has been described as a “duel over accuracy,” some believed that removing race from the equation would represent an unscientific concession to racial justice advocates while others felt that the choice to incorporate race as a binary without discussion of social nuance and genetic ancestry is what lacked scientific merit [15]. The work of the task force resulted in a 2021 peer-reviewed publication officially recommending the removal of the race variable from the equation [13, 15]. Subsequently, the Organ Procurement and Transplantation Network (OPTN) board voted to eliminate the use of race-based eGFR calculations when assessing candidacy for kidney transplantation and mandated the use of the new race-neutral eGFR 2021 CKD EPI Creatinine Equation effective July 27, 2022 [16, 17]. This change corrected the overestimation of renal function for Black patients and changed eligibility for transplant referral to be at the same point in kidney disease progression as patients of all other races.

For those Black patients already placed on the waiting list, the OPTN Minority Affairs and Kidney Transplantation Committees proposed a policy change that would require transplant centers to determine which patients could be eligible for a wait-time modification due to the transition from the race-inclusive to the race-neutral eGFR calculation [18, 19]. After this policy was approved and took effect on January 5, 2023, all transplant centers were required to (1) inform all patients on their waiting list of the policy change, (2) determine candidates who would be eligible for wait-time modifications, (3) submit applications for wait-time modification, and (4) attest that the review and modification application was completed [12, 18, 19]. Patients who were candidates for wait-time modification could apply for a new qualifying date of when they had a race-neutral eGFR ≤ 20 mL/min per 1.73 m^2^ versus a race-inclusive eGFR > 20 mL/min per 1.73 m^2^. Transplant centers were given from January 5, 2023, to January 3, 2024, to satisfy all four of these requirements [17, 18, 20, 21].

Analyses of the effects and reach of this policy change have not been well-studied. While initial reports suggest that many patients have received transplant wait-time back from these modifications, we intended to investigate the impact of this change and, more specifically, to determine whether all patients who stand to benefit from these changes have been reached [19, 20].

## Methods

### Setting

Northwestern Medicine (NM) eliminated the use of race-inclusive eGFR calculations on November 2, 2021. Given the OPTN mandate, the NM Comprehensive Transplant Center (CTC) dedicated personnel from January 2023 to January 2024 to process wait-time modification for eligible patients on the kidney transplant list. As the largest transplant center in the state of Illinois serving a diverse, metropolitan population, NM provides a rigorous setting for critically examining the reach of this policy with a focus on those who were not affected by the policy but perhaps should have been.

### Transplant Center Protocol

To satisfy the OPTN requirements, members of the CTC at NM first notified all patients by letter (regardless of race) of the new mandate to use a race-neutral eGFR calculation and the possibility for patients to gain wait-time back. Next, CTC employees reviewed a list of patients currently on the NM kidney transplant waiting list who self-identified as Black or African American upon listing, which was generated by the United Network for Organ Sharing (UNOS). CTC personnel (including quality improvement officers and transplant nurse coordinators) performed manual searches within the electronic medical record (EMR) to identify laboratory values that would qualify these patients for wait-time modification (i.e., an eGFR calculation in which a race-neutral eGFR was calculated to be ≤ 20 mL/min but a race-inclusive African American eGFR was reported as > 20 mL/min). Though multiple eGFR values can be present, the oldest qualifying value is identified and then sent to UNOS for verification. All patients on the NM transplant waiting list were then notified if they did and if they did not qualify for wait-time modification per the laboratory values that were available in the EMR. Patients were given the appropriate contact information to the transplant center if they felt that they were eligible for wait-time and/or an inappropriate determination had been made.

### Study Design and Data Collection

A retrospective EMR review was performed in April 2024 of all patients on the UNOS generated list of Black or African American patients on NM’s kidney transplant waitlist. Clinicodemographic information, including, date of birth, sex, clinical status (i.e., living or deceased), end stage kidney disease (ESKD) etiology, blood type, insurance type, level of education, and 5-digit home zip code, was recorded from the EMR. Original dialysis start time and adjusted dialysis start time after wait-time modification were recorded as reported on official communication from the transplant center to patients. The source of qualifying laboratory data was also noted as being from the listing date eGFR laboratory values or from maintenance dialysis.

The 2020 Area Deprivation Index (ADI) was used as a measure of social and structural determinants of health for each patient and was obtained using available 5-digit zip code data. ADI is a publicly available dataset maintained by the University of Wisconsin School of Medicine and Public Health [22, 23]. It is a score from 0 to 100 created from 17 characteristics of one’s social environment, including education (e.g., high school graduation rates), household characteristics (e.g., number of inhabitants per household), social support (e.g., single-parent households), and income [24]. This score provides a standardized ranking at the census tract level [24]. Population-weighted means of census tract ADI were calculated to assign each patient an ADI value based on 5-digit zip code. Patients were then categorized into the following groups based on their ADI ranking: C1/Least Deprived (1st ADI quintile 0–20), C2/Less Deprived (2nd ADI quintile 21–40), C3/Moderately Deprived (3rd ADI quintile 41–60), C4/More Deprived (4th ADI quintile 61–80), and C5/Most Deprived (5th ADI quintile 81–100) areas.

To minimize the risk of identification of individuals, clinicodemographic categories have been collapsed where possible and missing or unknown data have been omitted.

### Analysis

To compare patients who received time back to those who did not, chi-square tests were utilized for categorical variables and two-tailed student *t*-tests were used to compare numeric variables using STATA software (version 18.0, College Station, TX). This study was approved by the Institutional Review Board of Northwestern University (STU00220883).

## Results

### Time Gained Back

**Fig. 1:**
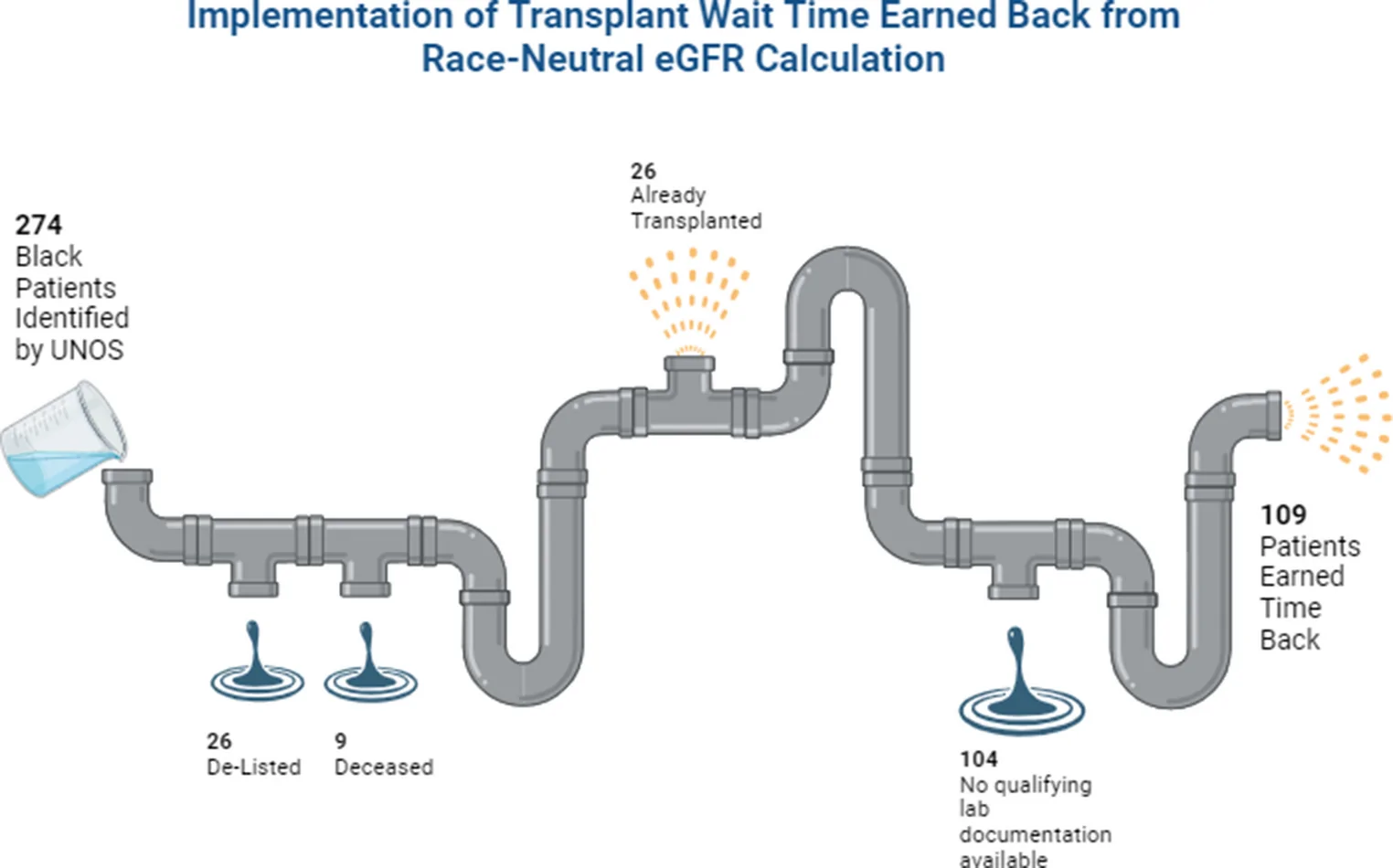
Depiction of proportion of patients to receive wait-time modification

Of the 854 total patients waitlisted during the first year of wait-time modifications at this center, 274 patients who were identified by UNOS as candidates for wait-time modification. Of these 274, 26 (9.5%) patients were delisted by the time that their candidacy was evaluated, nine (3.3%) patients were deceased, 26 (9.5%) patients had already been transplanted, and 104 (38.1%) patients did not have qualifying lab values that could be seen in the NM EMR system. As a result, 109 (39.9%) patients met the full criteria and successfully gained time back (Fig. 1). The mean and median time gained back were 1.68 years and 2.74 years, respectively. The minimum time received was 1 month and the maximum time received back was 21 years. Collectively, these 109 patients gained a total of 298.45 years of additional kidney transplant wait-time back due to the eGFR calculation policy change. As of July 15, 2024, 27 (24.8%) of these patients have gone on to receive a kidney transplant.

### Who Gained Time Back

**Fig. 2:**
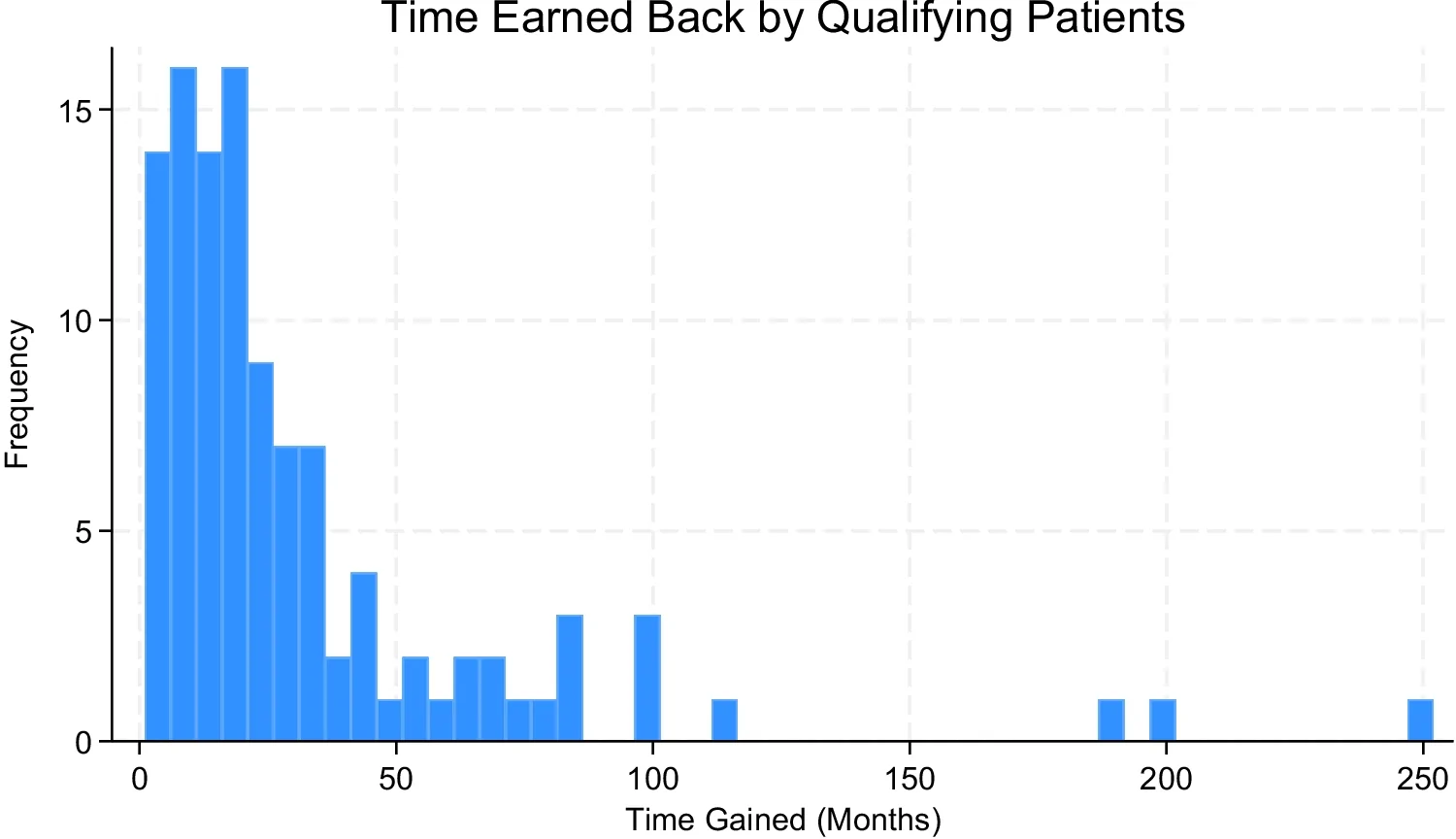
Histogram of wait-time gained back by qualifying patients

The mean age of the 109 patients who gained time back was 56 years old (SD 9.9) (Table 1). Forty-seven (43.1%) patients were female. At the time of chart review, four (3.7%) patients were deceased. Hypertension was the most common etiology of end-stage kidney disease (*n* = 33, 30.3%), followed by type 2 diabetes mellitus (32, 29.4%), other (e.g., cardiorenal syndrome, obstructive nephropathy, drug toxicity) (*n* = 15, 13.8%), and FSGS (*n* = 13, 11.9%). O was the most common blood type (*n* = 62, 56.9%), followed by type A (*n* = 25, 22.9%), and type B (18, 16.5%). Most patients who gained time back had public primary insurance (*n* = 62, 56.9%). Most of these patients reported that they were not working at the time of their evaluation (*n* = 56, 51.4%). 42.2% (*n* = 46) of patients reported that they had attended some college or trade school while 36.7% (*n* = 40) reported that high school was their highest level of education. Most patients who gained wait-time back were from moderately deprived (*n* = 40, 36.7%) and more deprived (*n* = 31, 28.4%) social environments (Fig. 2).

**Table 1:** Demographics of patients who did and did not receive wait-time modifications

	**Received time back**	**Did not receive time back**	**Total cohort**	***P*-value**
**Total**	109	165	274	
**Age, mean (SD)**	55.9 (9.85)	55.0 (11.35)	55.4 (10.77)	0.503
**Sex, *N* (%)**				0.854
Men	62 (56.9)	92 (55.8)	154 (46.2)	
Women	47 (43.1)	73 (44.2)	120 (43.8)	
**Status**				0.367
Living	105 (96.3)	153 (93.9)	258 (94.9)	
Deceased	4 (3.7)	10 (6.1)	14 (15.2)	
**Primary diagnosis**				0.122
Hypertensive nephrosclerosis	33 (30.3)	55 (33.3)	88 (32.1)	
Type 1 and type 2 diabetes mellitus	36 (33.0)	57 (34.5)	93 (33.9)	
Systemic lupus erythematosus	12 (11.0)	8 (4.9)	20 (7.3)	
FSGS	13 (11.9)	11 (6.7)	24 (8.8)	
Other	15 (13.8)	31 (18.8)	46 (16.8)	
**Blood type**				0.468
O	62 (56.9)	92 (55.8)	154 (56.2)	
A	25 (22.9)	30 (18.2)	55 (20.1)	
B	18 (16.5)	35 (21.2)	53 (19.3)	
AB	4 (3.7)	5 (3.0)	9 (3.3)	
**Insurance type, *N* (%)**				0.317
Public	62 (56.9)	95 (57.6)	157 (57.3)	
Private	47 (43.1)	65 (39.4)	112 (40.9)	
Missing	0 (0)	5 (3.0)	5 (1.8)	
**Education level**				0.167
Completed high school or less	43 (39.4)	61 (37.0)	104 (38.0)	
Attended college/trade school	46 (42.2)	61 (37.0)	107 (39.1)	
Associate/bachelor’s degree	15 (13.8)	20 (12.1)	35 (12.8)	
Post-college graduate degree	5 (4.6)	15 (9.1)	20 (7.3)	
**Employment status at evaluation**				0.322
Working	52 (47.7)	71 (43.0)	123 (44.9)	
Not working	56 (51.4)	88 (53.3)	144 (52.6)	
**ADI (continuous), mean (SD)**	54.8 (19.0)	56.0 (18.8)	55.5 (18.9)	0.628
**ADI (categorical)**				0.312
C1/least deprived	4 (3.7)	6 (3.6)	10 (3.7)	
C2/less deprived	21 (19.3)	32 (19.4)	53 (19.3)	
C3/moderately deprived	40 (36.7)	45 (27.3)	85 (31.0)	
C4/more deprived	31 (28.4)	68 (41.2)	99 (36.1)	
C5/most deprived	12 (11.0)	12 (7.3)	24 (8.8)	
**Source of lab data**				0.004
Listing EGFR	67 (61.5)	68 (41.2)	135 (49.3)	
Maintenance HD	36 (33.0)	87 (52.7)	123 (44.9)	
Other	6 (5.5)	10 (6.06)	16 (5.8)

### Who Did Not Gain Time Back

Among the 165 patients who did not gain wait-time back, the average age was 55 years (SD 11.4), the patients were predominantly men (*n* = 92, 55.8%), and the most frequent etiologies were hypertensive nephrosclerosis (*n* = 55, 33.3%) and type 2 diabetes mellitus (*n* = 47, 28.5%), similar to the cohort that gained additional wait-time. There were also no significant differences in blood type, insurance type, education level, and structural environment (ADI) between the two groups. In contrast to those who did receive time back, those who did not were less likely (41.2% vs. 61.5%) to have the listing date eGFR as the source of qualifying laboratory values. Rather, the non-qualifying patients tended to have bloodwork taken during maintenance dialysis as the source of their laboratory data (52.7% vs. 33.0%) (*p* < 0.001) (Table 1).

## Discussion

Black patients in the USA have had their access to kidney transplantation limited for decades due to the insidious effects of an eGFR calculation that incorporated race. Predicated on the false notion that Black patients have increased muscle mass and therefore may require a different calculation of renal function, a race-inclusive eGFR calculation systematically forced Black patients to wait longer before their kidney disease reached the threshold needed for a clinician to place a referral to a nephrologist or to a transplant center [25]. During the first year of wait-time modifications at the largest transplant center in the state of Illinois, 274 Black/African American patients were eligible for modifications (as of June 30, 2023), of which 109 gained back a total of almost 300 years of kidney transplant wait-time ranging from 1 month to 21 years with a median of 2.74 years per patient. This is even greater than the 1.9-year median delay estimated from predictive modeling prior to the policy change [26, 27]. Given that the National Kidney Foundation reports an average wait-time of 3–5 years on the transplant waitlist, 2.74 years is nearly all the waiting time needed to receive a deceased donor kidney transplant in this country, which would benefit patients by decreasing their time on dialysis and improving their quality of life [28, 29]. With 5-year survival on hemodialysis estimated to be 40.7%, these wait-time modifications undoubtedly saved the lives of Black patients at this transplant center and at centers around the country [8].

While removing race from the eGFR calculation was a critical first step, this work demonstrates that many patients have yet to reap the benefits of this evidence-based policy change. Only 39.8% of patients identified by UNOS as NM candidates for wait-time modification ultimately received wait-time back. This value lies in between the 26% and 54.3% reported by other single institution studies that have assessed similar data [19, 20]. Sadly, some patients died (*n* = 9) or were delisted (*n* = 26) during the 1-year period in which the transplant center identified and applied for these modifications. Of note, 26 patients (9.5% of patients identified by UNOS) were already transplanted by the time their eligibility for wait-time modification was considered. Importantly, the majority of the 165 patients who did not receive time back (*n* = 104) lacked qualifying laboratory data. This means that the transplant center was unable to identify lab results dated on or before their dialysis start date that had (1) a race-inclusive eGFR > 20 and an eGFR value < 20 for non-African American candidates or (2) a documented re-estimation of a patient’s GFR with a race-neutral calculation that showed an eGFR value < 20. It is likely that additional time could have been gained if those patients had received the appropriate lab draws earlier in their disease progression and if the transplant center had access to those records. There are multiple sources of laboratory data available to transplant centers, including (1) the eGFR data available from the date the patient was listed for transplant and (2) maintenance laboratory studies taken before or since they started dialysis. Patients who received wait-time back in this study were more likely to have qualifying laboratory data from their eGFR at listing as the basis for gaining more time. However, not all patients had both the African American eGFR and non-African American eGFR values from their listing eGFR available, necessitating the use of more recent labs from maintenance dialysis to assess eligibility.

Patients who consistently receive care for kidney disease or other chronic disease likely have basic metabolic panels checked in a variety of locations, including emergency departments, primary care offices, and independently owned diagnostic centers (e.g., Quest Diagnostics). However, the laboratory data from these locations are not centralized and may not be readily accessible in an individual transplant center’s EMR. While it is more common for ethnic and racial minorities to first learn of their kidney disease when they require emergent dialysis [30, 31], many Black patients with kidney disease will likely have laboratory results from a variety of sources. The challenge is in accessing these results. It is unsurprising that the most accessible eGFR result—the one that was used to place the patient on the transplant waiting list—was the most common source of qualifying laboratory values submitted to receive wait-time modification using the new eGFR calculation. However, casting a wide rather than a narrow net for qualifying laboratory results is precisely how the full restorative justice potential of this single policy change will be realized. Casting a wide net may include interventions such as creating dedicated patient navigators who guide patients through the process of requesting older lab data, creating scripts for patients to use when contacting dialysis centers or medical practices where labs were previously drawn, or requiring transplant centers to document their efforts to assist patients without lab data readily available. Evaluating the efficacy of any such interventions as well as attempts to standardize this process across centers could be the subject of future research.

Previous studies estimated that approximately 70,000 Black patients in the USA could stand to benefit from the transition to a race-neutral eGFR calculation [26, 32]. However, an April 2024 report released by the OPTN revealed that only 14,494 wait-time modifications were processed between January 2023 and January 2024. Reaching all the individuals who may have been inadvertently impacted by this policy cannot be a passive endeavor; broad reach requires the collective and intentional action by key stakeholders in the transplant process. Operationalizing this policy change may require additional effort from transplant center staff who are—unfortunately—already overworked and overburdened by high patient volumes. Undoubtedly, Black patients with ESKD are owed this effort given decades of unequal treatment, the years spent on dialysis in lieu of receiving a transplant, and the lives that were lost while waiting. As Mohottige and colleagues state, “we must demonstrate sustained, collaborative commitments to remove structural barriers and create a new paradigm for embedding equity in future policies” [26]. Helping patients to receive the time back that they are owed will require a sustained collaboration between patients, providers, and transplant centers. It will require advocacy on the part of nephrologists and primary care providers to help patients track down laboratory results from previous providers or dialysis centers. It will require empowering patients to know what laboratory values to look for in their personal records and how to navigate requests for information from prior offices. Unfortunately, there have been some documented reports of clinics and healthcare systems erasing old data that calculated eGFR using a race-inclusive calculation, which has clear implications for patients who are now trying to prove that they could have qualified for a transplant earlier [15]. This is a critical time period in which taking the extra effort to work with individual patients to find qualifying laboratory results could result in wait-time modifications that truly change their clinical trajectory. This moment of disentangling structural racism from the kidney transplant process can be leveraged to forge trust and collaboration between disenfranchised Black patients and the transplant centers that have largely existed just outside of their reach.

There is wide variation between centers regarding the process of implementing the race-neutral eGFR policy change and satisfying the OPTN requirements. Most centers describe an internal review of eligible patients that involves the review of available laboratory data [19, 20]. However, for some centers, this internal review involved engagement with referring nephrologists and primary care providers to provide education about the policy change and to communicate the importance of reviewing historical laboratory results [19]. Some centers describe ongoing conversations with patients about the importance of checking their personal records for results that can be used to increase wait-time [19]. While these practices are certainly admirable, they are not the standard. It is also possible, given the results reported here, that differences in rates of African American patients receiving wait-time back between centers may be due to different practices for reviewing charts for historical lab data. There is currently a lack of guidance around the specific protocol centers should follow to engage providers and patients in this process of wait-time modifications. Transplant centers were simply given until January 3, 2024 to have “assessed your waiting list, submitted modifications and supporting documentation, and notified candidates”[21]. Although the one-year mandate has passed, centers still have an obligation to notify newly registered kidney candidates about the possibility of earning additional time based on qualifying lab values and to continue to submit wait-time modifications for eligible candidates[21]. Therefore, the time is still ripe for centers to share the protocols that they have developed over this 1-year period and to collaboratively create a standardized process for patient and provider engagement to ensure that every single patient who can benefit from this policy change has the opportunity to do so.

This study contributes meaningfully to the literature by focusing on the patients who did not receive wait-time modifications after the race-neutral eGFR policy went into effect and by identifying lack of access to lab data as a key structural barrier in need of ongoing intervention. However, this study also has noteworthy limitations. The data reported and analyzed here represents one center’s experience with the eGFR policy change and wait-time modifications. While it is possible that other centers had a different experience with implementation of this policy, the CTC at NM is a large-volume transplant center that serves a diverse population of patients with ESKD seeking access to kidney transplantation. The number of Black patients who received time back at NM is greater than the number reported at other centers [20], indicating that the data is robust enough to provide valuable insight on the implementation process.

The OPTN’s decision to adopt a race-neutral eGFR calculation and to allow for wait-time modifications for those listed with a race-inclusive eGFR has changed the lives of Black patients with ESKD. At one center alone, 109 patients received a total of ~ 300 years of transplant wait-time with a median of 2.74 years gained back at the individual patient level. Twenty-seven of these patients have already received kidney transplants. However, with only 39.8% of Black patients who were candidates for additional time actually receiving time back, it is clear that the policy change alone is not enough. While individual transplant centers have reported about their experiences, a national study on the impact of missing lab data or an assessment of how patients have navigated requesting old lab values would help to characterize this structural barrier on a national scale. To address this barrier, collaboration is needed between transplant centers nationwide to standardize the implementation of this race-neutral eGFR policy and to maximize the ongoing potential for impact. Beyond reporting the patients who successfully receive wait-time modifications, national efforts could be directed toward providing active bureaucratic assistance to patients who may not know where or how to find old lab data. Advocacy for patients by providers, by the transplant centers, and by the entire transplant system is critical to realizing the full potential of this good faith effort to enable Black patients to have truly equitable access to kidney transplantation.

## Data Availability

The data that support these findings are available upon reasonable request to the authors.
